# How Boys Earn Money

**Published:** 1875-09

**Authors:** 


### HOW BOYS EARN MONEY.

We have been telling our young friends for some time, how they could acquire valuable knowledge and at the same time earn some money. Perhaps neither of these objects appear to them of much importance at present, but we know the time will come when they will thank us very cordially if we will but urge upon them the wise course.

When the writer of this paragraph was a boy, he was on friendly terms with the proprietor of a printing office. The good man gave him permission to assist about the office. He told a compositor to teach the little fellow "the case"—that is, to tell him in which of the numerous little compartments containing types, the various letters were to be found; whereabouts in the "upper-case" the capitals were, and where the "small-caps;" which box held a comma and which a period. In a few days this small boy could set type and distribute it; could "justify," that is, space out so that the lines were all of exact length; could lift out a stick full and place it on the galley with ease; and, in short, prided himself on being something of a printer.

Now although we never learned the printers' trade, and although we have not set a stick full of type for more than twenty years, we have never forgotten the lesson learned in our voluntary apprenticeship on odd days at the printing business. We then acquired a knowledge of the details of the art, as well as of literary work. proof-reading and the like, which has greatly aided us in our subsequent labors,—a knowledge which money would not buy for us, and which we should be very unwilling to part with at any price.

Not all boys have a friend in the printing business, who will take the trouble to instruct them in the details of his craft. But if this is denied them, other advantages exist which no boy had in the olden time. There are books which tell all that a boy needs to know about printing, and a very small amount of money will establish a printing office in the home of any boy Mr. Watson, of No. 53 Murray street, New York, and No. 73 Cornhill, Boston, manufactures beautiful and strong little presses, some of which are considerably larger than are needed for printing two pages of our JOURNAL at once—and some of which are only suitable for cards and small circulars, but upon all of which nice printing can be executed.

We know a little fellow of thirteen years, who has owned one of these presses for exactly two months. It was a present to him and cost $18. It is called a "Note Press," and prints a sheet five inches one way, by seven and a half inches the other,—about the size of the reading matter on this page. He has had no experience as a printer, yet he has cleared one dollar each working day since the press was carried into his room. At first he earned nothing; but on one occasion he cleared $2.50, and on another he made $2.75 in one day. He got an order for supplying a merchant with 5,000 fine Bristol cards, printed on both sides, at $3 a thousand, and completed the job in just one week. Here were 10,000 impressions, each one requiring the application of the ink-roller, the placing of a card in position, a pull upon the lever and the removal of the card. The work was done and very neatly done. The impressions were sufficiently dark, but there was no excess of ink, and there was no soiling of the cards by handling with dirty fingers or off-setting.

The 5,000 cards, No. 3, cost $4.25, and all the rest was net earnings—say $10.75 for a week's work. The great printing offices asked $20 for the same job, and could not have done it better to save their lives.

We mention this case to encourage the boys. If they have any desire to be and do something, let them begin. And we hope parents will remember that if they desire to make useful men and women of their boys and girls, they should encourage them to be useful, and to take an interest in useful subjects.

